# Stone Clearance by Computed Tomography after Percutaneous Nephrolithotomy: A Descriptive Cross-sectional Study

**DOI:** 10.31729/jnma.5219

**Published:** 2020-08-31

**Authors:** Chitaranjan Shah, Robin Bahadur Basnet, Arvind Shah, Prakash Chhettri, Anup Chapagain, Parash Mani Shrestha, Anil Shrestha

**Affiliations:** 1 Department of Urology, National Academy of Medical Sciences, Bir Hospital, Kathmandu, Nepal

**Keywords:** *computed tomography*, *fluoroscopy*, *percutaneous nephrolithotomy*, *residual fragments*

## Abstract

**Introduction:**

Percutaneous nephrolithotomy has the highest stone free rate among other procedures with relatively higher complication rate. Post-operative imaging after stone surgeries has not been uniform. This study was done to study about the stone clearance by computed tomography after percutaneous nephrolithotomy.

**Methods:**

The descriptive cross-sectional study was conducted in the Department of Urology, Bir Hospital for six months duration. The patients undergoing percutaneous nephrolithotomy and those with intra-operative fluoroscopic clearance were evaluated with a low dose computed tomography after 48 hours to assess residual fragments its size and location. Patient's demographics, stone characteristics and complications were compared between the stone free and with residual stone patients.

**Results:**

Out of 72 percutaneous nephrolithotomy performed, 40 patients were included in the study. Low dose computed tomography kidney, ureter and bladder after 48 hours of surgery detected residual fragments in 11 (27.5%) patients. The RFs size of <4mm were found in 7 (63.63%) of cases whereas RFs of >4mm were found in 4 (36.36%). The stone size was 352.47±97.47 mm^2^ and 254.79±172.68 mm^2^ in group with residual fragments and stone free group respectively.

**Conclusions:**

Low dose computed tomography kidney, ureter and bladder done for assessment of stone clearance after 48 hours of percutaneous nephrolithomy detected residual in around one fourth of patients, however majority of them had residual fragments <4mm. Intra-operative fluoroscopic clearance may over estimate stone clearance after percutaneous nephrolithomy as about one third of patients still may have residual fragments of >4mm size.

## INTRODUCTION

Primary aim of stone related surgeries is to achieve highest stone clearance with minimal or no morbidities.^1^ Percutaneous nephrolithotomy (PCNL) is considered a surgical modality with highest stone free rate with relatively higher complication rate.^2,3^ Post-operative imaging after stone surgeries has not been uniform. The heterogeneity of assessment is due to: modality of imaging used; X-ray alone, ultrasonography alone, X-ray and ultrasonography (USG) combined or Computed tomography (CT), timing of imaging and definition of stone free status: <2mm, <4mm or no fragments.^4–6^

Standard CT of kidneys, ureters and bladder (KUB) has sensitivity of 100% and specificity 95% for detection of residual fragments.^7^ Low dose CT KUB has a comparable sensitivity and specificity with the benefit of three times lower ionizing radiation exposure than standard CT KUB.^8,9^

So, the aim of this study was to find stone clearance after PCNL by low dose CT KUB.

## METHODS

The descriptive cross sectional study was conducted in the Department of Urology, National Academy of Medical Sciences (NAMS), Bir Hospital, Kathmandu, Nepal between November 2019 and April 2020. Ethical clearance from the Institutional Review Board was taken. Informed consents for the study were taken from all the patients. Total 72 cases of PCNL were done during the study period. Sample size was calculated using the formula:

$$\begin{array}{ll} \text{n} & \text{= }{\text{Z}}^{\text{2}}\times \text{(p}\times \text{q)}/e^{\text{2}}\\ & \text{= 1}{\text{.96}}^{\text{2}}\times (\text{0.05}\times 0.95)/{(\text{0.05)}}^{\text{2}}\\ & \text{= }72.9=\text{approximately 73 cases.} \end{array}$$

Where,

n = required sample size
p = prevalence of residual stones (5%)^14^
q = 1-p
e = margin of error, 5%
Z = 1.96 at 95% Confidence Interval

Pre-operative assessment with CT KUB was done for measurement of stone in two largest dimensions (mm^2^) and stone density measured in Hounsfield Unit. Sterile urine before the procedure was ensured for every patient. All the patients undergoing PCNL were included during the study period. The exclusion criteria were patient with age below 14 years, stone density less than 500HU, patients not giving consent, no fluoroscopic clearance and patients not undergoing CT scan post-operatively.

All PCNLs were done in prone position under spinal anesthesia. A transpapillary puncture was made with help of fluoroscopic guidance using 18 gauze two-part needles after retrograde opacification of the pelvicalyceal system via the ureteral catheter. The tract dilatation was done by single step dilatation technique. Stones were fragmented with pneumatic lithotripter. Stone fragments were removed either by continuous normal saline irrigation or with forceps. The exit strategies were total tubeless, tubeless or standard. Intraoperative variables studied included stone fragmentation time, total operative time, number and location of tracts and fluoroscopic clearance. Post PCNL after 48 hours, low dose CT KUB was performed to assess the stone clearance rate. Patients were grouped into “stone free” and with “residual stone”. RFs were assessed for size and location.

Statistical analysis was performed using the Statistical Package for Social Sciences version 23. All categorical data were expressed in absolute number and numerical continuous data were expressed in mean ±standard deviation.

## RESULTS

Out of 72 PCNLs, 40 patients were included in the study. Thirty-two patients were excluded because six were underage for study, no fluoroscopic clearance could achieve in nine cases, 10 patients had stone of less than 500 HU and CT KUB was not done at 48 hours in seven patients (Table 1).

**Table 1:** Basic characteristics of patients.

	Stone free n (%)	Residual stone n (%)
Total patients	29 (72.5)	11 (27.5)
Gender		
Male	17 (42.5)	6 (15)
Female	12 (30)	5 (12.5)
Stone location		
Pelvic	13 (32.5)	6 (15)
Upper pole	4 (10)	3 (7.5)
Mid Pole	1 (2.5)	0 (0)
Lower Pole	8 (20)	2 (5)
Pelvi-ureteric Junction	3 (7.5)	0 (0)
Dilatation of system		
None	4 (10)	0 (0)
Mild	15 (37.5)	2 (5)
Moderate	10 (25)	4 (10)
Severe	0 (0)	5 (12.5)

**Figure 1.**
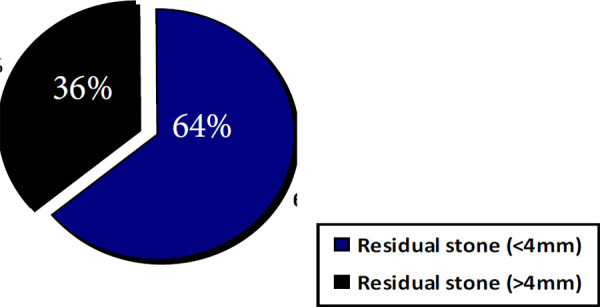
Distribution of residual fragments.

The mean age ±SD were 42.17±14.76 years in the “stone free” group and 38.67±12.25 years in the “residual stone” group. There were no significant differences in numbers of patients, sex distribution, age, location of stone and stone volume and between groups. Residual stone were detected in 11 cases (27.5%). The RFs size <4mm were found in 7 (63.63%) and >4mm in 4 (36.36%) of patients respectively (Figure 1).

**Figure 2.**
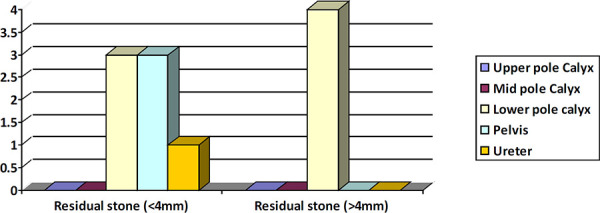
Distributions of residual stones across the calyces.

The mean stone size in “residual stone” group was 352.47±97.47 mm^2^ and in “stone free” group was 254.79±172.68 mm^2^. Stone density (mean±SD) was 941.77±333.32 HU and 992.08±338.82 HU in the “stone free” and “residual stone” groups respectively.

## DISCUSSION

Stone free rate is considered a surrogate marker of success of any renal stone surgery including PCNL.^10^ The possible reasons behind RFs are huge stone burden, stone migration, or stone fragments in an inaccessible calyx, termination of the procedure because of bleeding, complex anatomy increasing the technical difficulty, and inability to visualize the stone on fluoroscopy.^11^

A study in our center in 2018 showed stone free rate in 115 (73.24 %) patients out of 157 PCNLs, when X-ray KUB was done at the end of 4 weeks. Forty-two (27.75%) had residual fragments of more than 4 mm size, whereas in current study low dose CT KUB done after 48 hours showed fragments >4mm in 10% of cases only.^3^

Portis et al. used high magnification rotational fluoroscopy in conjunction with flexible nephroscopy to increase the intra-operative detection of residual stones. However 60% of patients were stone-free on postoperative day one CT KUB and 40 % of patients had residual stones 4 mm or smaller.^11^ In another study of Park et al. stone free rates of 62.3% and 20.8% were detected when x-ray KUB and CT KUB were used respectively at one month of PCNL. Unlike the current study the stone free rate was significantly low in CT KUB after one month of procedure.^12^

In this study, preoperatively majority of the stone were located in pelvis (50%) and lower pole (25%) and in about 60% of cases renal access were made through mid pole. Irrespective of that the 63.36% of RFs were detected in lower pole. It showed that one should have thorough inspection of the lower pole at the end of procedure even after the fluoroscopic clearance is achieved.

Ganpule et al. followed 2469 patients of PCNL with USG and x-ray KUB at 48 hours, one-month and three month for residual stones. The residual fragments were identified in 7.57% of the patients. Since X-ray KUB and USG over estimates the stone free rates by 17% to 35%, it has been mentioned that CT KUB is better in detecting RFs. The most common site for residual fragments was the lower calyx (57.7%). Similarly, in the current study the majority of RFs (63.63%) were in lower pole.^13^

Raman et al. evaluated 537 patients following PCNL with CT KUB and 42 (8%) patients had residual fragments. The majority RFs (47%) were in the lower pole. Sixty percent (25 of 42) of RFs were 2 mm or smaller and 79% (33 of 42) were smaller than 5 mm. Stone clearance with CT KUB in their study was significantly high in comparison to present study but the detected RFs size were comparable.^14^ Similarly, Atmoko et al. showed stone clearance of 62.6% only by CT KUB at one or two days after PCNL considering any diameter of stone as RFs.^15^

Stone free rate after stone surgery depends upon modality and timing of imaging used. Low dose CT KUB follow up after PCNL may detect higher RFs than X-ray KUB and USG. Though Low dose CT KUB has the highest sensitivity and specificity in detecting RFs, due to its cost and radiation hazards it is not done routinely for assessment of RFs postoperatively. There is no consensus and uniformity in timing of imaging used for the assessment of stone clearance after the stone surgery.

Single centered study, shorter duration of follow up and relatively smaller number of patients are the limitations of the study.

## CONCLUSIONS

Low dose CT KUB done for assessment of stone clearance after 48 hours of PCNL detected residual in around one fourth of the patients, however majority of them had residual fragments of < 4mm size. Intraoperative fluoroscopic clearance may over estimate stone clearance after PCNL as more than one third of those with residual fragments may still have fragments of > 4mm size. Studies with longer follow up duration are warranted to assess the significance of the residual fragments.

## Conflict of Interest

**None.**
